# Multidrug-resistant Tuberculosis in Central Asia

**DOI:** 10.3201/eid1005.030718

**Published:** 2004-05

**Authors:** Helen Suzanne Cox, Juan Daniel Orozco, Roy Male, Sabine Ruesch-Gerdes, Dennis Falzon, Ian Small, Darebay Doshetov, Yared Kebede, Mohammed Aziz

**Affiliations:** * Médecins Sans Frontières Aral Sea Area Programme, Uzbekistan and Turkmenistan, Tashkent, Uzbekistan; † National Reference Centre for Mycobacteria, Borstel, Germany; ‡ Ministry of Health, Nukus, Karakalpakstan, Uzbekistan; § Médecins Sans Frontières, Amsterdam, the Netherlands; ¶ World Health Organization, Geneva, Switzerland

**Keywords:** tuberculosis, drug resistance, MDR-TB, Central Asia, MSF, Uzbekistan, Turkmenistan

## Abstract

Multidrug-resistant tuberculosis (MDR-TB) has emerged as a major threat to TB control, particularly in the former Soviet Union. To determine levels of drug resistance within a directly observed treatment strategy (DOTS) program supported by Médecins Sans Frontières in two regions in Uzbekistan and Turkmenistan, Central Asia, we conducted a cross-sectional survey of smear-positive TB patients in selected districts of Karakalpakstan (Uzbekistan) and Dashoguz (Turkmenistan). High levels of MDR-TB were found in both regions. In Karakalpakstan, 14 (13%) of 106 new patients were infected with MDR-TB; 43 (40%) of 107 previously treated patients were similarly infected. The proportions for Dashoguz were 4% (4/105 patients) and 18% (18/98 patients), respectively. Overall, 27% of patients with positive smear results whose infections were treated through the DOTS program in Karakalpakstan and 11% of similar patients in Dashoguz were infected with multidrug-resistant strains of TB on admission. These results show the need for concerted action by the international community to contain transmission and reduce the effects of MDR-TB.

Tuberculosis (TB) has increased substantially in many parts of the former Soviet Union, particularly in those areas most affected by economic decline and failing health infrastructures (*1*). In addition to resurgent TB, significant proportions of multidrug-resistant TB (MDR-TB) have been demonstrated in small pockets where drug -susceptibility surveys have been conducted, and these areas have been termed “hot spots” (*2*,*3*). This article reports the results of a drug-susceptibility survey conducted by Médecins Sans Frontières (MSF) in collaboration with local ministries of health, in northwestern Uzbekistan and northern Turkmenistan in Central Asia. These regions, in addition to experiencing a substantial economic crisis, are facing a severe water shortage and the desiccation of the Aral Sea (*4*).

A TB treatment program based on the directly observed treatment strategy (DOTS) (*5*) recommended by the World Health Organization was introduced progressively into three regions south of the Aral Sea by the humanitarian medical aid organization, Médecins Sans Frontières. These regions, the Republic of Karakalpakstan, and Khorezm Oblast in Uzbekistan and Dashoguz Velayat in Turkmenistan (Figure, have a total population of approximately 4 million people. Implementation of DOTS started in 1998 and was completed with full coverage by late 2003. In 2002, more than 8,000 patients were registered and treated under DOTS from the three regions. Facilities for culturing sputum and drug susceptibility testing were not available in the area at the time of the survey.

**Figure:**
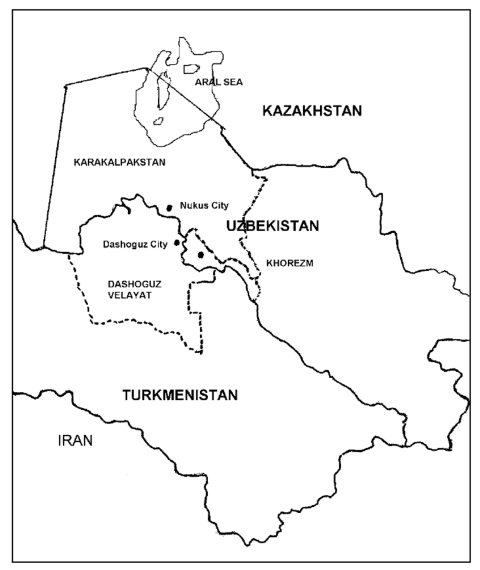
Aral Sea area, Uzbekistan and Turkmenistan.

From the start of DOTS implementation, >10% of those treated remained smear-positive. This finding, combined with a dearth of information on drug resistance in Central Asia, prompted the initiation of a drug-susceptibility survey with the aim of determining levels of resistance to both first- and second-line anti-TB drugs and, in particular, the levels of MDR-TB.

The current survey was conducted in two of the regions covered by DOTS, the Republic of Karakalpakstan in Uzbekistan and Dashoguz Velayat in Turkmenistan. Although adjacent, these regions are separated by an international border. Key characteristics of these regions and countries are given in Table 1; the two regions differ in terms of total case reporting rate and in economic status.

**Table 1:** Key characteristics of the two regions and countries included in the survey^a^




	Uzbekistan	Turkmenistan

Population (*6*)	25,256,000	4,834,000
GDP per capita (*6*)	U.S.$ 2,333	U.S.$ 5,269
Health expenditure per capita (*6*)	U.S.$ 86	U.S.$ 286
Estimated TB incidence rate (all cases) (*7*)	92/100,000/y	84/100,000/y
Estimated smear-positive case detection (*7*)	44%	68%
	Karakalpakstan	Dashoguz
Population (2001 estimated)^b^	1,549,761	1,041,372
DOTS implementation	1998 (pilot districts)–2003 (full coverage)	2000 (pilot districts)–2002 (full coverage)
DOTS case notification rate (all cases)	482/100,000/y	213/100,000/y
DOTS case notification rate (smear- positive cases)	192/100,000/y	89/100,000/y
% of smear-positive cases previously treated (2002)	55	53
Success rate (new smear-positive cases registered in 2001)	68%	82%


^a^GDP, gross domestic product; TB, tuberculosis; DOTS, directly observed treatment strategy.

^b^Source: ministries of health in each region.

## Methods

### Study Design

The survey was designed on the basis of recommendations given in the World Health Organization/International Union Against Tuberculosis and Lung Disease **(**WHO/IUATLD) guidelines for surveillance of drug-resistant TB (*8*). It was designed to be a cross-sectional survey of smear-positive pulmonary TB patients in whom DOTS TB treatment had been initiated in four districts in the Autonomous Republic of Karakalpakstan in Uzbekistan and four districts in Dashoguz Velayat in Turkmenistan. Three of the districts in each region were selected because they had the longest running DOTS programs in the region. The fourth district in each region was selected as the newest district implementing DOTS before initiation of the survey. When the survey was initiated, 7 of the 17 districts in Karakalpakstan were implementing DOTS TB treatment, as were 7 of the 9 districts in Dashoguz. Although selection of districts in which to conduct the survey was not random, all smear-positive patients whose TB was diagnosed in DOTS laboratories (regardless of previous TB treatment status) from July 2001 in the selected districts were sequentially requested to participate in the survey. Patients were excluded if they refused to undergo DOTS treatment or if they did not reside in one of the districts chosen in the study. Patients defined as “chronic,” that is, patients whose TB was not resolved after at least two courses of DOTS treatment, were not eligible to be included in the study. Smear positivity was defined as at least one sputum sample reading >10 bacilli/100 fields in a sputum smear by direct microscopy.

The objectives of the study were described to each patient, and written informed consent was obtained before an additional sputum sample was collected for the study. All patients were informed that the results would not be available to either themselves or their treating physician and would not affect their treatment. At the time of the survey, no opportunity was available for patients to be treated with second-line drugs under DOTS-Plus. To ensure patient confidentiality, sputum samples and clinical information forms were encoded with a unique survey identification number.

MDR-TB is defined as resistance to at least isoniazid and rifampin. On the basis of estimated levels of 10% and 20% MDR-TB for new and retreatment cases, sample sizes were calculated to be 81 for new cases (10%±6%, 95% confidence limit [CL]) and 100 for re-treatment cases (20%±7%, 95% CL). Because approximately 50% of patients with positive smears have been previously treated, a sample size of 100 was chosen for each category in both regions, for a total of 400 patients. Recruitment continued until the desired sample size was reached or surpassed in both categories and in both regions.

### Demographic and Clinical Information

Each patient consenting to participate in the survey was interviewed by the admitting doctor in each TB facility. A clinical information form was developed, based on recommendations by the WHO and IUATLD Disease (*8*), to collect basic demographic, socioeconomic, and medical information. Particular attention was paid to questions that would clarify previous TB treatment for each patient. Questions were included about previous hospitalization and about which anti-TB drugs the patient may have taken. Previous TB treatment assessed by the interview was then compared to the patient’s previous TB treatment status as determined through the functioning DOTS program. Any discrepancies were then investigated and clarified by reinterviewing the patients and physicians who treated their condition. In addition, for patients from Karakalpakstan only, medical records from the previous Soviet system of TB treatment were checked for previous TB treatment.

New patients were defined as those who had received no or <1 month of antituberculosis drug treatment before diagnosis. Retreatment patients were all those previously treated with TB drugs for >1 month. Data on the HIV status of patients with TB are not available in this area.

### Transport of Sputum Samples and Laboratory Testing

Because no local capability for culture and drug-sensitivity testing exists, sputum samples were transported directly to a supranational reference laboratory (SRL) in Europe. Sputum specimens were shipped from both regions separately either weekly or biweekly throughout the survey. Samples were first transported to the regional laboratories in each region by car. They were then flown to the capital cities in Uzbekistan and Turkmenistan; they were then transported as international air cargo to Frankfurt, Germany, and on to Hamburg and the SRL in Borstel, Germany. All international regulations pertaining to the transport of infectious material were followed.

Sputum samples were cultured, and drug-sensitivity testing was performed by the German SRL in Borstel. Strains were tested for drug sensitivity to the five first-line drugs used in the DOTS program in the Aral Sea area, namely, isoniazid, rifampin, pyrazinamide, ethambutol, and streptomycin. In addition, drug-susceptibility testing for three second-line drugs was performed; susceptibility to prothionamide was tested on all strains, whereas susceptibility to capreomycin and ofloxacin was tested on all strains, excluding the first 45 sent from both countries (24 from Uzbekistan and 21 from Turkmenistan, 11% of strains). Strains from a small, randomly drawn sample of patients with MDR-TB (20 isolates) and patients whose strains were susceptible to antimicrobial drugs (10 isolates) were later tested for resistance to kanamycin.

Primary isolation and culture of mycobacterial isolates followed standard recommendations, where specimens were decontaminated by NALC-NaOH and added to two solid media; Löwenstein-Jensen and Stonebrink ad MGIT960TB (Becton-Dickinson Microbiology Systems, Cockeysville, MD) (*9*). For all strains, drug-susceptibility testing was performed on Löwenstein-Jensen media by the proportion method. If growth was insufficient, drug-susceptibility testing was performed by using the modified proportion method in BACTEC 460TB (Becton-Dickinson). SRL participates in annual quality control programs within Germany and within the WHO and Centers for Disease Control and Prevention (CDC) SRL network. In addition, an internal quality control system for all techniques and media was used.

### Statistical Analyses

All clinical and laboratory data were entered into a database by using EpiInfo (6.04, CDC, Atlanta, GA). Chi-square analysis was used for comparisons of proportions. Logistic regression analysis was performed to identify variables independently associated with MDR-TB (SPSS version 10.0, SPSS Inc., Chicago, IL). Cluster sampling was taken into account through use of the Fleiss quadratic approximation in calculating 95% CL for the proportions of MDR-TB in the sample. Separate design effects were calculated for each region.

## Results

### Recruitment of Patients

Patient recruitment began in July 2001 in both regions and was completed by the end of January 2002 in Karakalpakstan and March 2002 in Dashoguz. In total, 441 patients were enrolled in the study, 213 from Dashoguz and 228 from Karakalpakstan. Overall, 76% of eligible patients were recruited in Dashoguz and 68% in Karakalpakstan. The main reasons patients were not enrolled were the following: refusal to participate (13% in Karakalpakstan and 3% in Dashoguz), patient default before treatment (8% in both Karakalpakstan and Dashoguz), and logistic constraints in the timely collection and transport of sputum samples (9% in Karakalpakstan and 2% in Dashoguz). Other reasons for nonparticipation in the study included the following: unable to produce an additional adequate sputum sample (5% in Dashoguz), patient imprisonment after diagnosis (3% in Dashoguz), and patient transfer out of the program after diagnosis. The relative biases introduced by these reasons for nonparticipation are unknown; however, the high rate of refusal to participate in Karakalpakstan was mostly due to inadequate patient communication in the early stages of the survey and is therefore unlikely to have affected the representativeness of the sample.

### Laboratory Culture Results

All 441 sputum samples were sent to SRL in Borstel, Germany. *Mycobacterium tuberculosis* was cultured from 416 samples (213 from Karakalpakstan and 203 from Dashoguz). Overall, 12 (2.7%) of the 441 samples were found to be contaminated, and an additional 11 (2.5%) could not be cultured. Other than *M. tuberculosis*, two other strains of *Mycobacterium* (one *M*. *bovis* and one *M*. *fortuitum*) were grown.

### Demographics of the Study Population

For the purposes of analysis, only patients for whom a valid culture for *M. tuberculosis* was obtained were included. The final sample consisted of 106 new patients and 107 retreatment patients in Karakalpakstan and 105 new cases and 98 retreatment cases in Dashoguz. Patients ranged in age from 11 years to 77 years, with a mean of 34 years and a median of 31 years. The mean age was slightly but nonsignificantly higher in retreatment patients. Overall, 60% of the patients in the study sample were male, with more men among retreatment cases than new cases (65% vs. 55%). Overall 53% of all patients with positive smears registered in the DOTS program in the year 2002 in Karakalpakstan and Dashoguz were male.

Considerably more single men than single women lived in both regions; overall, 19% of women and 40% of men were single. Approximately 70% of all patients, both men and women in both countries, reported at least 10 years of education. No women reported previous imprisonment, whereas 25% of men in Dashoguz and 36% of men in Karakalpakstan reported being in prison at some stage during their lives.

### First-line Anti-TB Drug Resistance

Results on resistance to the five first-line drugs tested are given in Tables 2 and 3. In Karakalpakstan, the strains of 52% of new patients and 20% of retreatment patients were fully sensitive to all five first-line drugs. In Dashoguz, strains from 70% of new patients and 38% of retreatment patients were sensitive to the five first-line drugs. The most notable monoresistance (resistance to just one first-line drug) was seen for streptomycin in both regions (in 11% of all Karakalpakstan patients and in 14% of Dashoguz patients). Similarly, streptomycin showed the highest levels of drug resistance overall; strains from 58% of all patients in Karakalpakstan and from 37% of patients in Dashoguz were resistant to streptomycin. Major resistance to isoniazid also occurred, with 53% of all patients in Karakalpakstan and 31% in Dashoguz infected with resistant strains. Resistance to rifampin was closely correlated to multidrug resistance; only one patient had a strain that was resistant to rifampin without also being resistant to isoniazid.

**Table 2:** First-line anti-tuberculosis drug resistance results, Karakalpakstan, Uzbekistan^a^




	No. new cases (%)	No. retreatment cases (%)	Total no. (%)

Total tested	106	107	213
Any resistance	51 (48.1)	86 (80.4)	137 (64.3)
Monoresistance			
H only	3 (2.8)	7 (6.5)	10 (4.7)
R only	0	0	0
E only	0	0	0
S only	12 (11.3)	11 (10.3)	23 (10.8)
Z only	0	1 (0.9)	1 (0.5)
H and R resistance			
MDR	14 (13.2)	43 (40.2)	57 (26.8)
HR only	0	1 (0.9)	1 (0.5)
HRE only	0	0	0
HRS only	4 (3.8)	10 (9.3)	14 (6.6)
HRZ only	0	0	0
HRES only	6 (5.7)	19 (17.8)	25 (11.7)
HREZ only	0	0	0
HRSZ only	1 (0.9)	0	1 (0.5)
HRESZ	3 (2.8)	13 (12.1)	16 (7.5)
H + other resistance			
HE only	0	0	0
HS only	13 (12.3)	15 (14.0)	28 (13.1)
HZ only	1 (0.9)	1	2 (0.9)
HES only	2 (1.9)	4 (3.7)	6 (2.8)
HEZ only	0	0	0
HSZ only	1 (.9)	3 (2.8)	4 (1.9)
HESZ only	5 (4.7)	1 (0.9)	6 (2.8)
R + other resistance			
RE only	0	0	0
RS only	0	0	0
RZ only	0	0	0
RES only	0	0	0
RESZ only	0	0	0
Any drug resistance			
Any H resistance	39 (36.8)	74 (69.2)	113 (53.1)
Any R resistance	14 (13.2)	43 (40.2)	57 (26.8)
Any E resistance	16 (15.1)	37 (34.6)	53 (24.9)
Any S resistance	47 (44.3)	76 (71.0)	123 (57.7)
Any Z resistance	11 (10.4)	19 (17.8)	30 (14.1)


^a^H, isoniazid; R, rifampin; E, ethambutol; S, streptomycin; Z, pyrizinamide.

**Table 3:** First-line anti-tuberculosis drug resistance results, Dashoguz, Turkmenistan^a^




	No. new cases (%)	No. retreatment cases(%)	Totalno. (%)

Total tested	105	98	203
Any resistance	32 (30.5)	61 (62.2)	93 (45.8)
Monoresistance			
H only	6 (5.7)	8 (8.2)	14 (6.9)
R only	0	1 (1.0)	1 (0.5)
E only	0	0	0
S only	16 (15.2)	13 (13.3)	29 (14.3)
Z only	0	0	0
H and R resistance			
MDR	4 (3.8)	18 (18.4)	22 (10.8)
HR only	0	0	0
HRE only	0	0	0
HRS only	3 (2.9)	7 (7.1)	10 (4.9)
HRZ only	0	0	0
HRES only	1	6 (6.1)	7 (3.4)
HREZ only	0	0	0
HRSZ only	0	3 (3.1)	3 (1.5)
HRESZ	0	2 (2.0)	2 (1.0)
H + other resistance			
HE only	0	1 (1.0)	1 (0.5)
HS only	5 (4.8)	10 (10.2)	15 (7.4)
HZ only	0	1 (1.0)	1 (0.5)
HES only	1 (1.0)	6 (6.1)	7 (3.4)
HEZ only	0	0	0
HSZ only	0	3 (3.1)	3
HESZ only	0	0	0
R + other resistance			
RE only	0	0	0
RS only	0	0	0
RZ only	0	0	
RES only	0	0	0
RESZ only	0	0	0
Any drug resistance			
Any H resistance	16 (15.2)	47 (48.0)	63
Any R resistance	4 (3.8)	19 (19.4)	23
Any E resistance	2 (1.9)	15 (15.3)	17
Any S resistance	26 (24.8)	50 (51.0)	76
Any Z resistance	0	9 (9.2)	9


^a^H, isoniazid; R, rifampin; E, ethambutol; S, streptomycin; Z, p, pyrizinamide.

Levels of MDR-TB were high; overall, 27% (95% CL 15% to 42%) of all positive smears from patients starting DOTS treatment were infected with MDR-TB strains in Karakalpakstan and 11% (95% CL 7% to 17%) in Dashoguz. The proportion of MDR-TB was higher in Karakalpakstan than in Dashoguz for both new and retreatment cases; 13% (95% CL 4% to 35%) versus 4% (95% CL 1% to 11%) for new cases and 40% (95% CL 21% to 62%) versus 18% (95% CL 11% to 28%) for retreatment cases.

### Second-line Anti-TB Drug Resistance

Resistance results to the three second-line drugs first tested are shown in Table 4. Of these three drugs tested on most strains, clinically significant resistance was shown only to prothionamide, with 16% resistance among MDR-TB cases in Karakalpakstan and 9% in Dashoguz. Kanamycin was tested on a randomly drawn sample of 20 previously identified MDR-TB isolates and 10 non-MDR-TB isolates from two districts in Karakalpakstan. Two of the MDR-TB isolates showed resistance to kanamycin (10%), whereas none of the 10 non-MDR-TB strains showed resistance.

**Table 4:** Second-line drug resistance results^a^

	New		Retreatment		MDR-TB^b^	
	Total	Resistant (%)	Total	Resistant (%)	Total	Resistant (%)
Karakalpakstan (Uzbekistan)						
Prothionamide	106	7 (7)	107	11 (10)	57	9 (16)
Capreomycin	88	1 (1)	101	1 (1)	56	1 (2)
Ofloxacin	88	2 (2)	101	4 (4)	56	1 (2)
Dashoguz (Turkmenistan)						
Prothionamide	105	1 (1)	98	4 (4)	22	2 (9)
Capreomycin	89	0	93	0	21	0
Ofloxacin	89	0	93	0	21	0

^a^All strains were tested for prothionamide; a representative subset of these were tested for both capreomycin and ofloxacin. ^b^MDR-TB, multidrug-resistant tuberculosis.

### Factors Associated with MDR-TB

To investigate factors associated with the high rates of MDR-TB, a multivariable logistic regression analysis was conducted with MDR-TB as the dependent variable. The following factors were entered into the model: sex, previous TB treatment, previous imprisonment, unemployment, alcohol use, and region. Factors significantly predicting MDR-TB in the model were previous TB treatment, region, and female sex (Table 5). Although a large proportion of male patients reported previous imprisonment, this factor was not significant on either univariate or multivariable analysis.

**Table 5:** Factors associated with MDR-TB in univariate and multivariable logistic regression analysis^a^




Odds ratios (95% confidence limits)	Univariate analysis	Logistic regression

Previous TB treatment	4.5 (2.6 to 8.0)	5.1 (2.8 to 9.3)
Region (Karakalpakstan)	3.0 (1.8 to 5.1)	3.6 (2.0 to 6.4)
Female gender	1.3 (0.8 to 2.2)	2.0 (1.1 to 3.7)
Unemployment	2.3 (1.1 to 5.1)	2.1 (0.9 to 4.8)
Previous imprisonment	1.7 (0.9 to 3.0)	1.3 (0.6 to 2.8)
Alcohol use	0.9 (0.4 to 2.3)	0.7 (0.3 to 1.9)


^a^MDR-TB, multidrug-resistant tuberculosis.

Since previous TB treatment is the strongest factor associated with MDR-TB, the logistic regression was repeated, including only new patients from both regions. Both female gender (OR 7.8, 95% CL 1.7 to 36.3) and region (OR 3.8, 95% CL 1.2 to 12.4) remained significant predictors of MDR-TB.

Clearly, the most notable factor predicting MDR-TB is previous TB treatment. Most retreatment patients in both regions reported only one previous episode of treatment, 60% in Karakalpakstan and 66% in Dashoguz. In addition, most retreatment patients were not previously treated under DOTS, 83% in Karakalpakstan and 75% in Dashoguz. Of those that received previous DOTS treatment, 50% were new patients before their previous DOTS treatment. These patients had levels of MDR-TB similar to retreatment patients overall (Karakalpakstan, 3/8, 38%; Dashoguz, 2/15, 13%). Those that received DOTS treatment in addition to previous non-DOTS TB treatment had higher rates of MDR-TB (Karakalpakstan, 6/8, 75%; Dashoguz , 7/14, 50%).

## Discussion

This survey has shown extremely high rates of MDR-TB in regions of two countries in Central Asia, although the confidence intervals are large because of the small sample size and cluster sampling. These findings, along with similar data from other regions, suggest that the former Soviet Union is one large hot spot for MDR-TB.

Because of logistic constraints, sampling was restricted to four districts implementing DOTS in each of the two regions. These districts were not randomly selected. However, as sampling was sequential, the final sample is representative of patients with positive smears whose cases were diagnosed in these districts. The DOTS strategy was implemented in the Aral Sea area, starting in the districts most affected by the environmental degradation. These districts are more economically deprived and therefore may have higher rates of TB incidence. However, we have no reason to suspect that the prevalence of drug resistance is different in neighboring districts. Drug resistance may well increase as patients are increasingly able to purchase drugs privately and use them sporadically. In addition, patients who refused DOTS treatment and those defined as having chronic TB were not included in the survey. These patients are more likely to have had previous erratic TB treatment and therefore may be more likely to harbor drug-resistant strains. If this were the case, then the figures presented here are an underestimate of the situation.

In this survey, careful attention was paid to the differentiation of new and retreatment patients. A retrospective review of pre-DOTS medical records in Karakalpakstan showed only one misclassified patient, among the 213 included. As well, in the Aral Sea area DOTS program, all patients in whom active TB is diagnosed have the opportunity to receive DOTS treatment, regardless of previous treatment status. Thus, no motivation or incentive exists for patients or doctors to misrepresent their previous TB treatment status, as has been suggested elsewhere (*10*). Nevertheless, some misclassification is possible but is not expected to greatly alter the high proportions of MDR-TB seen among new patients, particularly in Karakalpakstan. These levels of MDR-TB indicate the likely transmission of multidrug-resistant strains.

Testing showed some second-line drug resistance, particularly for prothionamide, among MDR-TB strains. The regions in our study are poorer and more isolated than other parts of both countries and other areas in the former Soviet Union, which possibly spares them from the high rates of second-line drug resistance seen in other areas (*11*). Although not all strains were tested for second-line resistance, the sample is still representative because only the first 11% of strains (Karakalpakstan and Dashoguz, respectively) from patients sequentially recruited into the survey were not tested for capreomycin and ofloxacin, and no systematic differences were found between the strains from first patients and strains from latter patients.

The difference in levels of TB drug resistance between the two regions that are geographically adjacent but separated by a national border provides clues regarding the emergence of drug resistance. Since gaining independence from the former Soviet Union, most Central Asian states have experienced a substantial decline in healthcare services (*12*). In Uzbekistan, during the 1990s, the gross domestic product (GDP) declined considerably as has the percentage of GDP spent on health (*13*). Turkmenistan fared somewhat better financially because of its considerable oil and gas wealth; it has a per capita GDP almost double that of Uzbekistan (*6*), with a relatively stable percentage spent on health (*14*).

These declines in healthcare spending in both countries have resulted in intermittent shortages of most first-line anti-TB drugs. Local TB physicians in Karakalpakstan (Uzbekistan) estimate that before the DOTS program started, nearly 50% of patients had their treatment interrupted because of problems with drug supply. Additionally, because of the lack of drugs, patients were often requested to purchase drugs themselves after they left the hospital for the continuation phase at home. Many patients likely could not afford all drugs and therefore purchased what they could, resulting again in treatment interruptions. High streptomycin resistance attests to the widespread use of this popular injectable antimicrobial agent, often as a short monotherapy course. Although drug shortages have been reported over the last decade in Turkmenistan, they likely affected an overall lower percentage of patients, which explains the lower rate of drug resistance seen in Dashoguz.

The extent to which drug resistance existed before the collapse of the Soviet Union is unknown. The Soviet system hospitalized TB patients for long periods, with consequently high levels of interruption and default. In addition, treatment regimens using combinations of all first-line and some second-line drugs were not standardized (*1*). These conditions may have contributed to a baseline level of resistance from which the notable level of MDR-TB shown here has emerged. Most retreatment patients were not previously treated under DOTS; thus, our results cannot be attributed to the implementation of DOTS.

Elsewhere in the former Soviet Union, high rates of MDR-TB have been seen among prisoner populations (*15*). Although a high proportion of patients in our study reported previous imprisonment, no greater level of MDR-TB was seen among these patients. This finding suggests that MDR-TB is not confined to specific sectors of the population, such as prisoners, but is a problem affecting the general community. Of particular concern in this area is the high rate of out-migration attributable to worsening environmental and socioeconomic conditions (*16*; unpub. data, Médecins Sans Frontières, 2002), which can lead to international transmission of MDR-TB.

The finding of a greater risk for MDR-TB among women, independent of previous TB treatment status, is important and confirms similar findings in Archangels Oblast in Russia (*17*) and Estonia (*18*). In the Aral Sea area, women make up slightly less than 50% of all patients with positive smears registered in the DOTS program. This statistic suggests a greater susceptibility to drug-resistant TB and warrants further research.

Clearly, such high rates of MDR-TB as seen in both Karakalpakstan, Uzbekistan, and Dashoguz, Turkmenistan, are a substantial threat to TB control. Standardized treatment with first-line drugs implementing the DOTS strategy would be expected to result in poor outcomes for more than one fourth of patients with positive smears in Karakalpakstan (*19*) and would render the WHO target of 85% success unattainable. These patients will remain infectious for long periods, with the resultant risk of transmitting drug-resistant strains. WHO suggests that high levels of MDR-TB (>3% among new cases) warrant the direct management of MDR-TB to contain transmission and reduce the high incidence and costs of this disease (*20*).

DOTS treatment on its own may well stop the production of more MDR-TB, but it is unlikely to reduce high levels of existing drug resistance (*21*). Effective treatment of all cases of TB is required to prevent transmission. MDR-TB treatment is expensive and lengthy, and the pool of those with expertise treating MDR-TB is limited. A simpler, more affordable, and more effective treatment strategy is required; however, until this exists, patients require treatment with existing strategies. As a result of this survey, Médecins Sans Frontières has decided to launch a pilot DOTS-Plus MDR-TB treatment project in Karakalpakstan because the cost of inaction will be high.
